# Heterogeneity at the invasion front of triple negative breast cancer cells

**DOI:** 10.1038/s41598-020-62516-8

**Published:** 2020-04-01

**Authors:** Koh Meng Aw Yong, Peter J. Ulintz, Sara Caceres, Xu Cheng, Liwei Bao, Zhifen Wu, Evelyn M. Jiagge, Sofia D. Merajver

**Affiliations:** 1 Department of Internal Medicine, Hematology/Oncology University of Michigan Medical School, Ann Arbor, 48109 USA; 2 Evelyn M Jiagge is currently at the Henry Ford Cancer Institute/Henry Ford Health System, One Ford Place, Detroit, Michigan USA; 3 Present Address: University of Michigan, Department of Urology, Ann Arbor, 48109 USA; 4 Present Address: Department of Physiology, School of Animal Medicine. University Complutense of Madrid, Madrid, 28040 Spain

**Keywords:** Cancer models, Breast cancer, Cell invasion, Breast cancer

## Abstract

Identifying better predictive and prognostic biomarkers for the diagnosis and treatment of triple negative breast cancer (TNBC) is complicated by tumor heterogeneity ranging from responses to therapy, mutational burden, and clonal evolution. To overcome the gap in our understanding of tumor heterogeneity, we hypothesized that isolating and studying the gene expression profile of invasive tumor cell subpopulations would be a crucial step towards achieving this goal. In this report, we utilized a fluidic device previously reported to be capable of supporting long-term three-dimensional growth and invasion dynamics of cancer cells. Live invading and matched non-invading SUM149 inflammatory breast cancer cells were enriched using this device and these two functionally distinct subpopulations were tested for differences in gene expression using a gene expression microarray. 305 target genes were identified to have altered expression in the invading cells compared to the non-invading tumoroid cells. Gene ontology analysis of the gene panel identified multiple biological roles ranging from extracellular matrix reorganization to modulation of the immune response and Rho signaling. Interestingly, the genes associated with the invasion front differ between different samples, consistent with inter- and intra-tumor heterogeneity. This work suggests the impact of heterogeneity in biomarker discovery should be considered as cancer therapy increasingly heads towards a personalized approach.

## Introduction

Despite the technological advances of molecular diagnostics, little progress has been made with respect to the development of robust biomarkers for diagnosis and treatment of triple negative breast cancer due to a high degree of tumor heterogeneity within and between samples. While next generation sequencing approaches have been successful in detecting biomarkers, a disconnect between the biomarkers and functional actionability remains^1^. To bridge such a disconnect, correlating the identified putative biomarkers to a functional role in disease progression, such as the tumor’s physical “invasion front” represents a suitable strategy toward tackling tumor heterogeneity. Invasion is also a critical measure of disease progression supported by clinical knowledge that the overall five-year survival rates for organ-confined epithelial cancers are generally high but drop once invasion occurs to regional sites. Therefore, understanding the invasion process represents an opportunity to catch the disease at an early stage and help develop biomarkers which improve clinical diagnosis of early invasiveness and potentially identify treatment strategies to improve clinical outcome^2^.

A multifactorial process, cancer cell invasion requires cells to undergo a phenotypic transition triggered by intrinsic genetic and/or extrinsic environmental factors. Intrinsic factors include the genetic mutations or epigenetic dysregulation while extrinsic factors include cellular (immune or stromal cells) or non-cellular (extracellular matrix stiffness and interstitial pressure)^2–5^. This phenotypic transition results in cancer cells first degrading and invading past the basement membrane either collectively or as single cells into adjacent tissues or regional sites^6^. The tumor invasion front is characterized by leader cells that appear to pave the way for other cancer cells to follow^2^. Specific biological processes such as Rho signaling and epithelial mesenchymal transition (EMT) transcription programs have been widely reported to play important roles in invasion and metastasis, although recent reports have suggested that EMT may not be as crucial as previously thought^7–9^. Regardless of the molecular mechanism driving invasion, invading cells located in the tumor invasion front constitute an area of research interest, as they likely possess a functional phenotype of clinical relevance. Being able to capture and analyze these unique invading subpopulations in the act of doing so, together with their matched non-invasive counterparts, will be key in making progress towards understanding and treating cancer. While much work has been done in this field, some of these studies compare between different patients or utilize cell lines of different invasive properties^10–14^, which may be confounded by existing inter-tumor heterogeneity. While there have been other reports studying spatial heterogeneity within patient samples, these studies utilized the use of formalin fixed primary tumor tissue for the identification of genomic markers^15,16^. These studies mainly focus on identifying genomic mutations as markers of invasion as the deleterious effects of formalin on RNA integrity has been well documented and although technological advances in processing has resulted in improvements in nucleic acid quality, time of fixation plays a crucial role in the process, making such approaches inconsistent when studying gene expression^17,18^. In this study, we adopted a different approach toward understanding the heterogeneity existing within TNBC, by using a previously reported fluidic device to enrich for live cells found within the invasion front of this disease subtype for the specific purpose of studying the gene expression profile associated with invasion^19^. This device allows the long-term culture of cancer cells as a tumor mass (termed tumoroid) and has been demonstrated to enable the tumor invasion front formation and analysis in prostate cancer cells. Using this device, live invading cells at tumor invasion front, along with the matching non-invading tumoroid mass of TNBCs were isolated and analyzed for differences in gene expression.

## Results

### Tumoroid culture of established or patient derived xenograft TNBC cell lines demonstrate different invasion dynamics in the fluidic device

**Figure 1:**
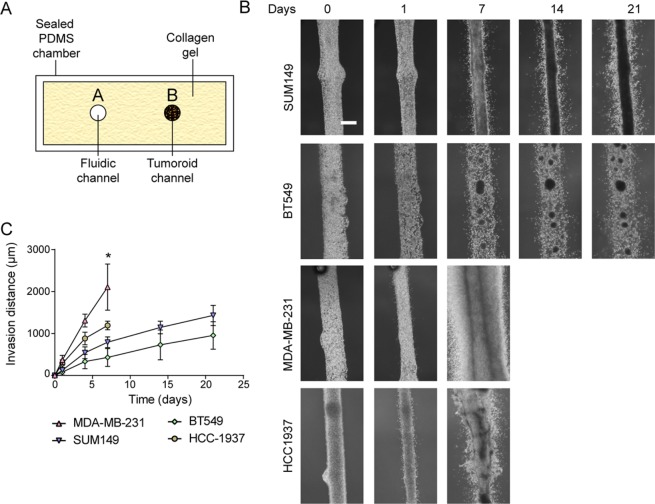
Fluidic culture of different breast cancer cell lines. (**A**) Schematic of fluidic device with tumoroid (side view). Cancer cells are seeded into one of the fluidic channels (channel B) and the channel subsequently sealed. Growth media is introduced into the chamber via peristaltic pump through channel A. (**B**) Phase images of tumoroid channel for triple negative breast cancer cell lines: SUM149 (first row); BT549 (second row); MDA-MB-231 (third row) and HCC1937 (last row). (**C**) Measurements of observed invasion distance of SUM149, BT549, MDA-MB-231 and HCC1937 over time. n = 3 *p < 0.05.

To determine the ability of the device to culture breast cancer cells, four triple negative breast cancer cell lines (SUM149, MDA-MB-231, BT549 and HCC1937) as well as three triple negative breast cancer patient derived xenografts (PDX1 (9040), PDX2 (vari004), and PDX3 (vari068)) were seeded and cultured in the fluidic device. Cancer cells were seeded into one channel while growth media was perfused into the system via the adjacent channel (Fig. 1A). Over a period extending to three weeks, varying rates of invasion into the surrounding collagen gel were observed in all samples tested. In terms of maximum measured invasion distance over a period of 7 days, MDA-MB-231 cells were the most invasive, with cells invading approximately 2 mm into the surrounding collagen within one week of culture. In contrast, HCC1937 cells were less invasive, invading only 1 mm by one week of culture before tumoroid culture became unstable. SUM149 invaded 1 mm by three weeks while BT549 invaded only 750 μm by three weeks of culture (Fig. 1C, Supp. Videos 1–4). This observation is in line with previous reports using Boyden invasion assays that demonstrated MDA-MB-231 to be more invasive than SUM149. Of note, SUM149 were noted to be less invasive than BT549 in that report^20^. Furthermore, the ability to form a tumoroid as well as the invasion front differed between cell lines. SUM149 consistently formed a defined tumoroid that appeared confined within the fluidic channel throughout culture, while MDA-MB-231 did not (Fig. 1C, Supp. Video 2). BT549 formed multiple smaller tumor spheroids within the middle channel while HCC1937 culture was unstable after one week, with noticeable deformation of the fluidic channel bearing the cancer cells (Fig. 1C). Single cell invasion was observed in MDA-MB-231, SUM149, HCC1937, and BT549 although collective invasion was also observed in HCC1937(Fig. 1C, Supp. Video 3).

The invasion rate of three PDXs also demonstrated a diversity of profiles. Among the three PDXs tested, PDX2 was the most invasive, with observed invasion into the surrounding collagen one day after seeding (Supp. Fig. 1, Videos 5, 6).

### Gene expression analysis yielded a panel of 305 genes to be affected during SUM149 invasion

**Figure 2:**
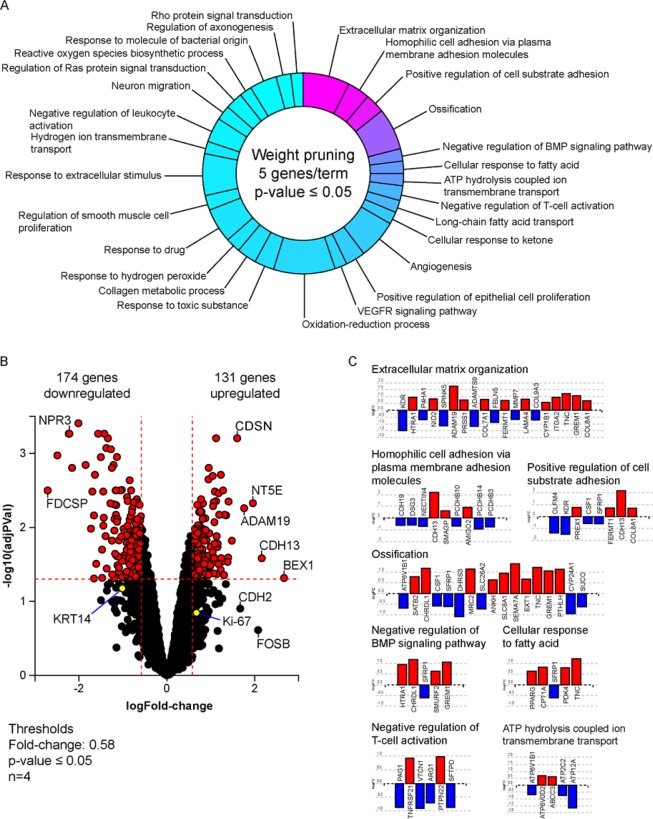
Differences in gene expression between invading and non-invading subpopulations. (**A**) Pie chart distribution of biological processes predicted to be affected during invasion based on gene expression profiling. Processes are arranged according to statistical significance, from magenta (lowest) to cyan (highest p-value), using weight pruning method of p-value correction. Processes were defined by a minimum of 5 genes per term with a p-value < 0.05. (**B**) Volcano plot of genes affected during invasion, displaying expression change (x-axis) in log2FC values with significance of the change (y-axis) represented in terms of the negative log (base 10) of the p-value (more significant genes are plotted higher on the y-axis). Cut-off thresholds in dotted lines were set at log 1.5-fold change in expression with adjusted p-value < 0.05. (**C**) Expression profile of target genes associated with several of the most significant predicted biological processes with the lowest p-values. Images were generated by the iPathwayGuide software (Advaita Bioinformatics, Ann Arbor, MI).

To discern the overall gene expression profile of invading cells, we first examined the invasive cancer cell subpopulation of SUM149. These invasive cells, visually determined by cells that advanced outside of the tumoroid into the collagen, were enriched along with the matching non-invading cancer cells in SUM149 tumoroid masses using a combination of enzymatic and mechanical dissociation. RNA was isolated from these two phenotypically distinct subpopulations and the gene expression profiles of these matched subpopulations were compared using an RNA expression microarray. From the microarray analysis, 305 genes were found to be differentially expressed at the invasion front compared to the non-invading tumoroid mass out of a set of 12,074 genes with measured expression (Fig. 2B). Principal component analysis of the different subpopulations suggested that the enriched invading subpopulation are distinct from the non-invading tumoroid subpopulation (Supp. Fig. 2). Examples of statistically significantly differentially expressed genes include cadherin 13 (CDH13) with a 2.1 log2-fold expression increase; brain expressed X-linked 1 (BEX1) with a 2.7 log2-fold expression increase, and a disintegrin and metalloprotease domain 19 (ADAM19) with a 1.8 log2-fold expression increase. The full set of relative gene expression measurements was further analyzed using the iPathwayGuide (Advaita Bioinformatics, Ann Arbor, MI) to help elucidate the possible biological roles encompassed. Results suggest that the tumor invasion front of SUM149 cell line is associated with specific regulation of with multiple biological roles including organization of the extracellular matrix and the negative regulation of leukocyte activation (Fig. 2A, Table 1). Interestingly, the expression of cytokeratin 14 (KRT14) which had a −0.9 log2-fold expression decrease, cadherin 2 (CDH2) which had a 1.9 log2-fold increase in expression, and Ki-67 which had 0.7 log2-fold expression increase, genes which have been reported to be associated with the invasion front, were not among the statistically significant targets identified^21^ (Fig. 2B). In addition, several target genes such as secreted frizzled-related protein 1 (SFRP1) and tenascin C (TNC) were associated with more than one biological process (Fig. 2C).

**Table 1:** Summary of Gene Ontology (GO) Biological Process terms identified.

goId	goName	countDE	countAll	ratio (DE/All)	pv_weight
GO:0030198	extracellular matrix organization	19	191	0.1	0.00002
GO:0007156	homophilic cell adhesion via plasma membrane adhesion molecules	9	51	0.18	0.000027
GO:0010811	positive regulation of cell-substrate adhesion	8	42	0.19	0.000043
GO:0001503	ossification	17	199	0.09	0.00038
GO:0030514	negative regulation of BMP signaling pathway	5	27	0.19	0.00141
GO:0071398	cellular response to fatty acid	5	30	0.17	0.0023
GO:0099131	ATP hydrolysis coupled ion transmembrane transport	5	30	0.17	0.0023
GO:0050868	negative regulation of T cell activation	6	51	0.12	0.00525
GO:0015909	long-chain fatty acid transport	5	32	0.16	0.0054
GO:1901655	cellular response to ketone	5	41	0.12	0.00914
GO:0001525	angiogenesis	16	237	0.07	0.00998
GO:0050679	positive regulation of epithelial cell proliferation	8	95	0.08	0.01038
GO:0048010	vascular endothelial growth factor receptor signaling pathway	5	45	0.11	0.01345
GO:0055114	oxidation-reduction process	25	405	0.06	0.01502
GO:0009636	response to toxic substance	8	103	0.08	0.01882
GO:0032963	collagen metabolic process	8	79	0.1	0.01982
GO:0042542	response to hydrogen peroxide	6	61	0.1	0.02195
GO:0042493	response to drug	14	205	0.07	0.02234
GO:0048660	regulation of smooth muscle cell proliferation	6	72	0.08	0.02632
GO:0009991	response to extracellular stimulus	17	215	0.08	0.02697
GO:1902600	hydrogen ion transmembrane transport	5	30	0.17	0.02705
GO:0002695	negative regulation of leukocyte activation	10	82	0.12	0.03308
GO:0001764	neuron migration	6	62	0.1	0.03354
GO:0046578	regulation of Ras protein signal transduction	7	82	0.09	0.03844
GO:1903409	reactive oxygen species biosynthetic process	5	50	0.1	0.03866
GO:0002237	response to molecule of bacterial origin	11	171	0.06	0.04151
GO:0050770	regulation of axonogenesis	6	68	0.09	0.04315
GO:0007266	Rho protein signal transduction	5	63	0.08	0.04909

Gene ontology ID (goId) and name (goName) are indicated in the first two columns respectively.

The third column (countDE) indicates the number of genes identified in the microarray that have been shown to be correlated with the biological process while the fourth column (countAll) indicates the total number of genes known to be correlated with the biological process. The fifth column shows the ratio of detected genes to all genes and the last column shows the corrected p-values.

**Figure 3:**
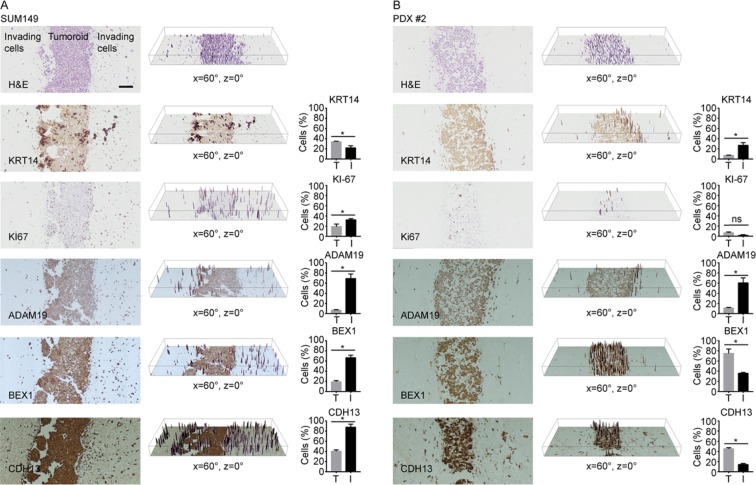
Immunohistochemistry of tumoroids for markers of invasion. SUM149 (**A**) and PDX #2 (**B**) tumoroids were stained for KRT14, Ki67, ADAM19, BEX1 and CDH13. 3D surface plots of staining intensity (represented in the middle panel of each row) while quantification of staining intensity in tumoroid (T) or invading cells (I) indicated as the percentage of cells that display stronger staining (represented in the third panel of each row). n = 3 *p-value < 0.05; ns = not significant.

To examine if the protein levels of several target genes at the tumor invasion front were also affected, whole SUM149 tumoroids were fixed, sectioned and stained for several target genes that demonstrated the highest fold change in expression (ADAM19, BEX1 and CDH13) along with cytokeratin 14 (KRT14) and Ki-67. In SUM149 tumoroids, cells that demonstrate stronger KRT14 staining appear localized to the interface between the tumoroid and collagen gel, while KRT14 staining appeared weaker overall in both the tumoroid interior as well as in the invading cells (Fig. 3A). Scoring of KRT14 staining, demonstrated a 50% increase in KRT14 staining in the tumoroid compared to invading cells. Unlike KRT14, Ki-67 positive cells were found both within the invasive subpopulation as well as the interface of the tumoroid and collagen with significantly fewer Ki-67 positive cells at the tumoroid interior. Quantification of Ki-67 staining supported this observation with the percentage of Ki-67 positive cells at the invasion front were 1.5-fold higher than those in the tumoroid (interface and interior) (Fig. 3A). In agreement with the microarray results, ADAM19, BEX1 and CDH13 protein levels were also higher within invading SUM149 cells compared to the tumoroids (5-, 3-, and 2-fold, respectively) as indicated by the higher percentage of cells that displayed stronger staining in the invading cells.

### Immunohistochemistry of PDX tumoroids indicate inter and intra-tumor heterogeneity

To examine if a common distribution of gene expression exists in other cells, tumoroids of cell lines BT549, HCC1937 and two TNBC PDXs (PDX1, PDX2) were stained for KRT14, KI-67, ADAM19, BEX1, and CDH13. In BT549, the percentage of cells that exhibited stronger KRT14 and ADAM19 protein levels were higher in the invading cells (2- and 1.5-fold, respectively) while the percentage of KI-67 positive cells was approximately 1.5-fold lower in the invading cells. While the percentage of cells that exhibited stronger BEX1 staining was 1.5-fold higher in invading cells, the change was not statistically significant. Likewise changes in CDH13 expression of 1.3-fold lower in the invading cells, were not statistically significant (Supp. Fig. 3).

In HCC1937 tumoroids, there was no statistical difference in percentage of cells positive for KI67 staining between the invading and non-invading tumoroid cells. There was also no significant difference in the percentage of cells that demonstrate stronger BEX1 between invading and tumoroid non-invading cell subpopulations. While the percentage of cells with stronger ADAM19 and KRT14 staining was higher in invading cells, the results were not statistically significant. However, the percentage of cells staining for stronger CDH13 expression was 2-fold higher in the invading cell subpopulation. (Supp. Fig. 3).

In PDX1, there were insufficient invading cells observed in the fixed samples for statistical analyses of IHC staining, although observable differences in staining could be seen with stronger staining for ADAM19, BEX1, and KRT14 at the interface of the tumoroid and collagen (Supp. Fig. 4).

In PDX2, the percentage of cells with stronger KRT14 and ADAM19 staining were higher (3- and 4-fold, respectively) while BEX1 and CDH13 were lower in invading cells (2- and 3-fold, respectively). While the percentage of cells positive for KI-67 was lower in invading cells, this difference was not statistically significant (Fig. 3B).

## Discussion

The initial objective of this report was to demonstrate the ability of our tumoroid culture device to identify potential gene signatures for the invading cells of triple negative breast cancers that could be used for diagnostic or therapeutic purposes. A strategy involving a prior enrichment step of different live subpopulations based on functional phenotype (such as invasive capability) followed by transcriptome analysis was adopted to reduce the complexity and heterogeneity that would have arisen, had such global analyses been carried out without prior enrichment. Using a novel fluidic device previously shown to be capable of maintaining long-term tumoroid growth and invasion dynamics, two functionally different, live subpopulations of cancer cells from a single sample could be enriched based on invasive capability for comparative downstream transcriptome analysis. This process was successful largely due to the ability of the device to establish sufficient spatial resolution between the invading and non-invading subpopulations. Moreover, the ability to isolate matching invading and non-invading subpopulations from a single device enabled the use of paired statistical analyses for each sample. Coupled with the capacity to correlate these gene expression signatures with a visually quantifiable functional behavior such as invasion highlights the advantage of the fluidic system.

Based on transcriptome analysis, 305 genes were identified to be up or down-regulated at a statistically significant level within the invading SUM149 cell subpopulation. Given that both invading and non-invading subpopulations were subjected to collagenase, it is unlikely that these differences in gene expression were an artifact of collagenase treatment. Interestingly, several known genes associated with invasion such as KRT14, cadherin 2 (CDH2) and FOSB, were not among the statistically significant targets^21–23^. In contrast to prior work on leading invasion edge biology in breast cancer, which showed KRT14 to be upregulated at the invasion front^21^, our gene expression microarray indicated a log 2-fold decrease of 1.03 in KRT14 (pval 0.077) within the invading SUM149 cells. Although this finding at the transcript level was not statistically significant, IHC staining for KRT14 confirmed the downregulation of KRT14 protein within invading cells in SUM149 tumoroids. Interestingly, within the tumoroid itself, KRT14 staining was observed to be strongest at the interface between the tumoroid and collagen, which suggests a possible association of KRT14 expression during the transition towards invasion. More work is needed to elucidate this possibility.

Pathway analysis of the differentially expressed genes between invading and non-invading cells using iPathway guide suggests that invading cells located at the tumor invasion front may play multiple roles that contribute to the progression of disease. Several of the biological processes identified were known to be associated with invasion, such as remodeling the extracellular environment, cellular adhesion, and Rho protein signal transduction^7^. The detection of these known processes coupled with the visual determination of invasion suggests that enrichment of the invading cell subpopulation was successful. Moreover, this work has identified several pathways beyond what is known to be associated with invasion. Of particular interest were the “negative regulation of T-cell activation” and “response to drug”. While more work is needed to extend these findings, the data presented suggest that the invasion process may contribute more to disease progression than just to the physical spread of disease.

While a candidate invasive gene signature was identified for SUM149; not surprisingly, given the heterogeneity of TNBC, we were unable to verify that this signature is common between the different cell lines tested in this study. This suggests strong inter-tumor heterogeneity in gene expression at the invasion front between different triple negative cells. While changes such as the upregulation of ADAM19 in the invading subpopulation may be shared among the triple negative breast cancer cells tested in this study, other genes, such as BEX1 and CDH13, were seen in some samples but not in others. This finding reaffirms the uniqueness of any individual biological sample and highlights the significance of considering inter-tumor heterogeneity when evaluating biomarkers. This work shows the potential utility of this device in discerning invasion front signatures, provided a large number of samples are analyzed in future studies. Future work will involve examining and comparing in detail gene expression profile differences between patients using more sensitive transcriptomic methods such as RNA-sequencing. In conclusion, our study suggests the importance of isolating and analyzing for each sample, the various cancer cell subtypes that are distinguished by differences in invading phenotypes. This will be an important consideration as in the era of tailored personalized therapy.

## Materials and Methods

### Cell culture methods

All cell lines underwent strict procedures to determine their purity and identity. SUM149 cells were obtained from Dr. Stephen Ethier. They were cultured in F12/DMEM media supplemented with 5% FBS, insulin hydrocortisone and penicillin/streptomycin. Cells were placed in a 37 °C tissue culture incubator at 10% CO_2_. Growth media was changed every other day. HCC1937 and MDA-MB-231 cells were grown in RPMI media supplemented with 10% FBS and penicillin/streptomycin. BT549 cells were grown in RPMI media supplemented with 10% FBS and insulin. HCC1937, MDA-MB-231 and BT549 cells were placed in a 37 °C tissue culture incubator at 5% CO_2_. Cells were subcultured at a 1:6 ratio upon attaining confluent culture. All cell lines were identified by short tandem repeats (STR) within approximately 10 passages of use.

### Patient derived material

Collection and use of human tumor tissue were conducted under approved IRB protocols at the University of Michigan and animal studies were performed under approved institutional animal care and use committee (IACUC) protocols. All three PDXs (PDX1: 9040; PDX2:vari004; PDX3:vari068) were obtained from the primary tumors of triple negative breast cancer patients who had signed informed consent. The PDXs vari068 and 9040 underwent targeted panel next-generation sequencing (NGS) and all 3 PDXs were developed *de novo* in our lab and were used at early passages (<10). The PDXs are stable in culture with regards to receptor expression and p53 status. The loss-of-function mutations have been ascertained by a targeted gene panel for the PDXs used in this study. PTEN homozygous loss-of-function variants were observed in 5 out 6 samples; p53 mutation are present in vari068.

Additional clinical history and description of the PDX patient donors can be found in Supplementary Table 1. Collected human tumor tissue from operation room was implanted into eight weeks old NSG mice at once when received. Breast tumor tissue was implanted into the mammary fat pad. Tumor was monitored once a week. Tumor was harvested when size reach at 0.6–0.7 cm. Tumor was cut into small pieces of 2–4 mm. The equipment and enzymes for the following procedures are from Miltenyi Biotec. Tissue pieces were transferred into gentleMACS C tube (cat# 130-096-334) containing 4.7 ml of RPMI1640 and enzyme mix (cat# 130-095-929). The tumor tissue was enzymatically digested using enzyme mix and mechanical dissociated using gentleMACS Dissociator. Total time of procedure is one hour. Between each turn of the gentleMACS Dissociator, the sample was incubated for 30 minutes at 37 °C under continuous rotation using the MACSmix tube rotator. After dissociation, the sample was sequentially filtered by a 70 µm and 40 µm strainer (Fisher # 22363548 & 22363547) to remove any remaining large particles from the single-cell suspension. Cell number was determined. The cell pellet was resuspended in 80 µl of 0.5% BSA/PBS and 20 µl of mouse cell depletion cocktail (cat# 130-104-694) per 10^7^cells. The sample was incubated in the 4 °C refrigerator for 15 minutes. Cells were magnetically separated with an LS column (cat# 130-042-401). The flow-through of enriched human tumor cells was collected and used.

### Tumoroid culture

Fluidic devices were constructed as previously described^10^. Two fluidic channels were molded the experiments described here. For devices bearing breast cancer cell lines SUM149, BT549, HCC1937, and MDA-MB-231, 1 × 10^5^ cells were injected into one of the fluidic channels to form the tumoroid. For PDXs or pleural effusion, 1 × 10^6^ cells were injected to form the tumoroid. Growth media used was 1 × DMEM supplemented with glucose, glutamine and 10% FBS. Tumoroids bearing HCC1937, BT549, and MDA-MB-231 were grown under 37 °C, 5% CO_2_, while tumoroids bearing SUM149, PDXs or pleural effusion were grown under 37 °C, 10% CO_2_. Phase images were taken of the tumoroids to monitor distance invaded by each of the cell lines. Tumoroids were cultured for up to three weeks or when invasion reached 1 mm on either side of the tumoroid mass. The selection of three weeks as an endpoint was based on the observation that most cell lines tested had demonstrated invasion by then. The distance 1 mm was selected as an endpoint due to the physical location of the fluidic channel delivering growth media.

### Separation of invading cells from non-invading tumoroid subpopulation

Invading cells were isolated from the non-invading tumoroid subpopulation using a combination of enzymatic and mechanical dissociation. First, the top PDMS layer of the fluidic chamber is peeled away to expose the collagen gel. Under a stereomicroscope, any surrounding excess collagen gel was first excised from the tumoroid. After mechanically removing as much collagen as possible, the tumoroid is further subjected to enzymatic treatment using collagenase A (Roche) to digest away the collagen surrounding the tumoroid and release any invading cells in proximity to the tumoroid. Collagenase A digestion was performed as suggested by the manufacture using a 0.25% w/v concentration of collagenase A solution resuspended in 1 × PBS and incubated at 37 °C. The digestion is visualized at five-minute intervals using brightfield microscopy to ensure over-digestion does not occur. After sufficient digestion has occurred, the tumoroid is removed carefully using a pair of sterile forceps and placed in a separate tube for RNA isolation. The collagenase A solution containing digested collagen and invading cells was centrifuged at 200 g for five minutes to harvest the invading cells for RNA isolation.

### Microarray analysis

RNA was first isolated from cells using RNeasy kit (Qiagen). The isolated RNA from different subpopulations were sent to the University of Michigan DNA Sequencing Core for low-input processing followed by Affymetrix GeneAtlas Human Gene 2.1.ST. To ensure consistency and reduced bias, the same amount of RNA from invading and non-invading subpopulations were subjected to low-input processing. Microarray data was further processed using Bioconductor (R) and ‘affycoretools v.1.50.6’. Gene expression measurements were generated using a robust multi-array average (RMA) modeling approach, and probe sets with variance less than 0.04 were removed. The PCA plots were generated using the plotPCA method of the affy library.

Differentially expressed genes were identified by fitting linear models using limma, selecting genes with a fold change threshold of 1.5 and an adjusted p-value of 0.05. Pathway analysis of the SUM149 cell line was performed using the commercial software iPathwayGuide (Advaita Bioinformatics, Ann Arbor, MI), which implements both an overrepresentation analysis and an impact analysis for pathway identification^24,25^. In this experiment, 305 differentially expressed (DE) genes were identified out of a total of 12,074 genes with measured expression from the microarray. These were identified using a threshold of 0.05 for statistical significance (p-value) and a log2 fold change of expression with absolute value of at least 0.585. These data were analyzed in the context of pathways obtained from the Kyoto Encyclopedia of Genes and Genomes (KEGG) database^26^, and gene ontologies from the Gene Ontology Consortium database^27^.

### Immunohistochemistry

Harvesting the tumoroids and immunohistochemistry were performed as previously described^19^. Briefly, PDMS devices were first disassembled and the tumoroids harvested and fixed in 4% paraformaldehyde fixative for 3 days. The tumoroids were then embedded in paraffin, sectioned, and stained by the Unit for Laboratory Animal Medicine *In-vivo* Animal Core at the University of Michigan. Hematoxylin and eosin (H&E) staining was performed and immunohistochemical (IHC) staining for KRT14 (BioLegend), Ki67 (Abcam), BEX1 (ThermoFisher) CDH13 (ThermoFisher) and ADAM19 (ThermoFisher) was conducted with hematoxylin counterstain. Non-invading tumoroid cells can be identified on the slides by a continuous mass of cells with a distinct straight border which mirrors the brightfield images of the live culture (Fig. 1B) while invading cells are indicated by single cells either rounded or elongated that are not part of the continuous mass of cells. To quantitatively represent the difference in protein levels between invading and non-invading cells, images of both populations in a single file are taken. 3D surface plot of these images was performed using the 3D surface plot application in ImageJ (NIH) to detect the staining intensity in invading and non-invading tumoroid cells. The number of cells in either subpopulation that exhibit stronger staining intensity are enumerated and further calculated as a percentage of all cells in the corresponding subpopulation.

## Supplementary information

Supplementary Dataset.

Supplementary Information.

Supplementary Video 1.

Supplementary Video 2.

Supplementary Video 3.

Supplementary Video 4.

Supplementary Video 5.

Supplementary Video 6.
