# Supplementary material for: Heterogeneity at the invasion front of triple negative breast cancer cells
Source: Sci Rep. 2020 Apr 1;10:5781. doi: 10.1038/s41598-020-62516-8 (PMC7113246; doi:10.1038/s41598-020-62516-8)
Supplement: Supplementary file 1 — Supplementary Dataset. [file 41598_2020_62516_MOESM1_ESM.pdf]

Supplementary Information

Supplementary videos can be found online

Heterogeneity at the invasion front of triple negative breast cancer cells

Koh Meng Aw Yong[1,ǂ], Peter J. Ulintz[1], Sara Caceres[1,#] Xu Cheng[1], Liwei Bao[1], Zhifen Wu[1], Evelyn M. Jiagge [2] Sofia D. Merajver[1,*]

[1] Department of Internal Medicine, Hematology/Oncology University of Michigan Medical School, Ann Arbor, 48109; U.S.A.

ǂ Koh Meng Aw Yong is currently at University of Michigan, Department of Urology, Ann Arbor 48109, U.S.A.

Sara Caceres is currently at Department of Physiology, School of Animal Medicine. University Complutense of Madrid, Madrid, 28040, Spain.

[2] Evelyn M Jiagge is currently at the Henry Ford Cancer Institute/ Henry Ford Health System, One Ford Place, Detroit, Michigan.

* Corresponding author (smerajve@umich.edu)

Disclosures: The fluidic device used in this study for tumoroid culture has been licensed to K.A. Patent pending on the fluidic device used in this study.

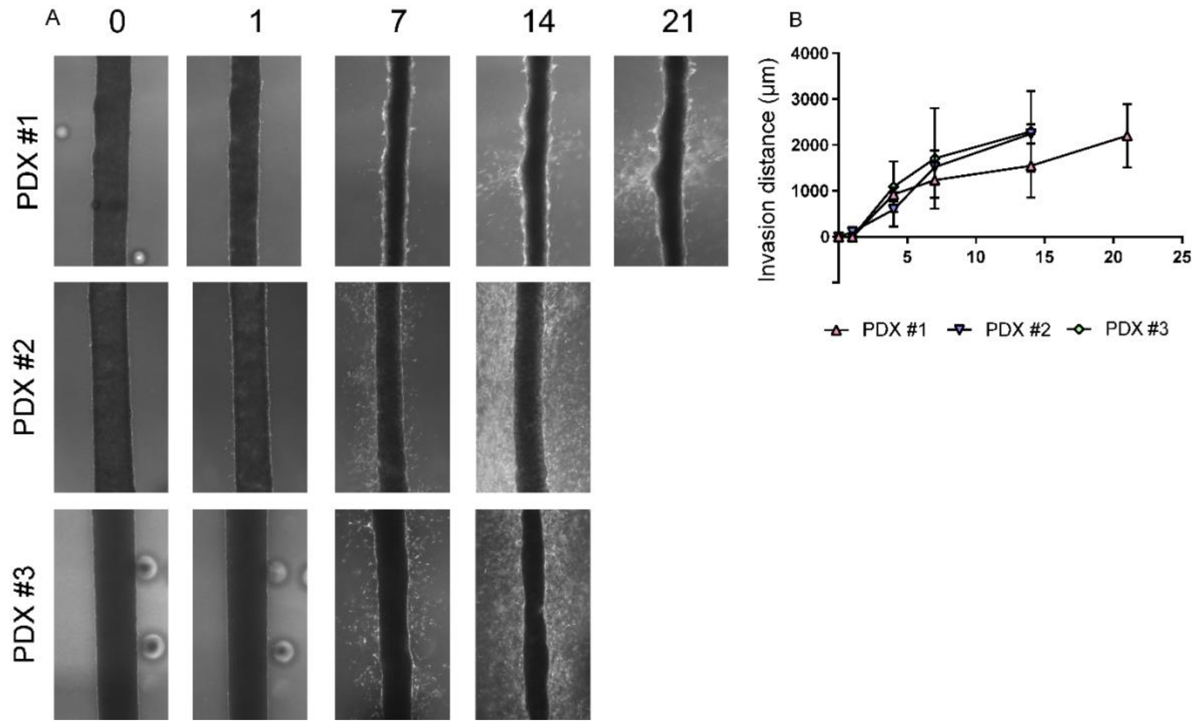

SUPPLEMENTARY FIGURES AND CAPTIONS

Supp. figure 1. Fluidic culture of different patient derived xenografts, pleural effusion, and cell lines. **A.** Phase images of tumoroid channel and invasion for triple negative breast cancer PDXs: PDX #1 (top row); PDX #2 (second row) PDX #3 (last row). **B.** Measurements of observed invasion distance of PDXs #1-3 over a time period of 21 days.

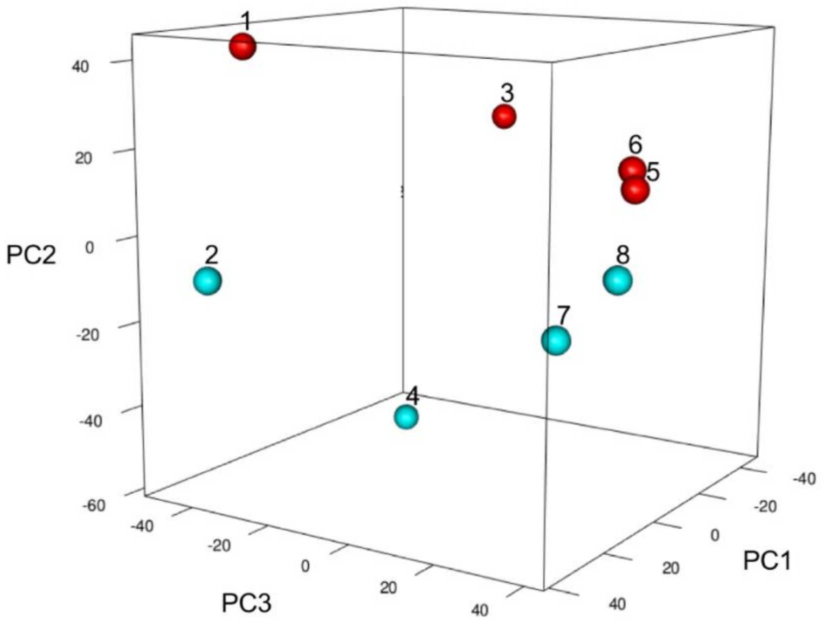

Biological replicate #	Invading #	Matched Non-invading #
1	2	1
2	4	3
3	7	5
4	8	6

Supp. figure 2. Three-dimensional principal component analysis of invading and non-invading SUM149 subpopulations. In total, four SUM149 replicates were analyzed with each replicate consisting of paired invading and non-invading subpopulations. Invasive and non-invasive subpopulations demonstrate separation along the second dimension.

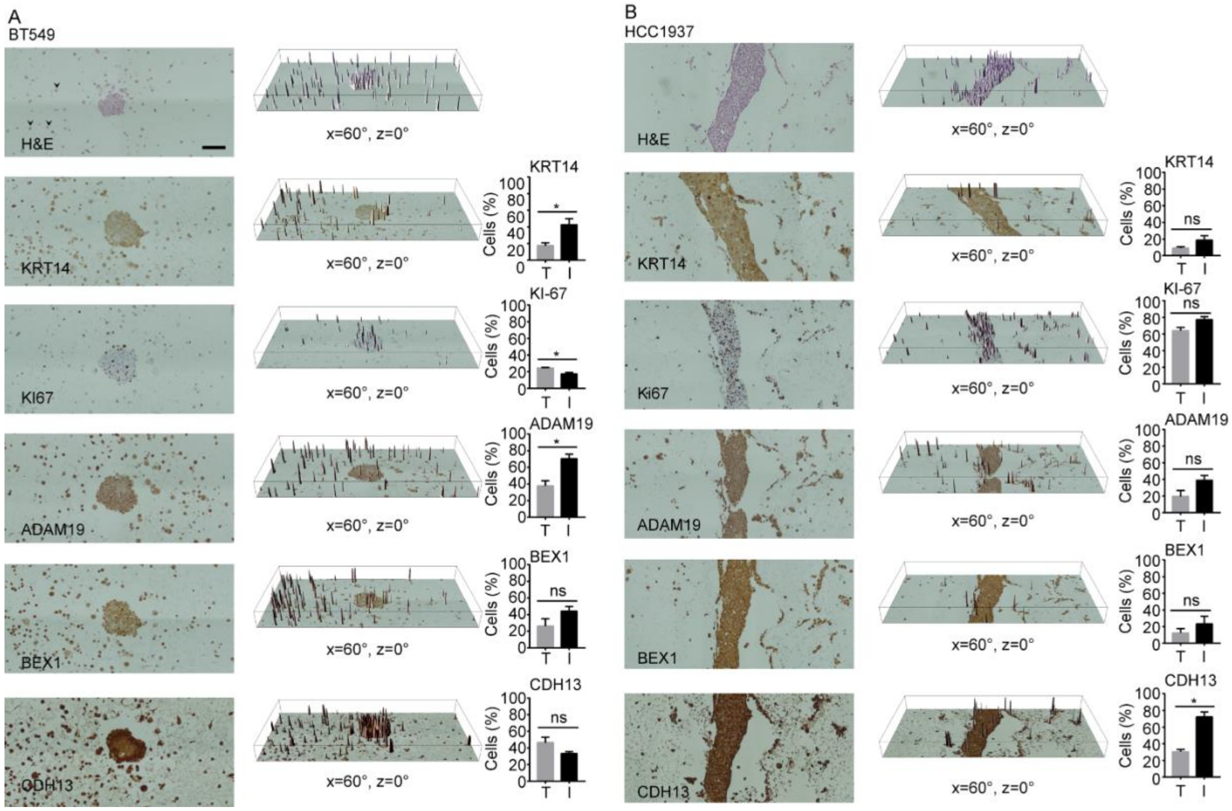

Supp. figure 3. Immunohistochemistry of tumoroids for markers of invasion. BT549 (A) and HCC1937 (B) tumoroids were stained for KRT14, Ki67, ADAM19, BEX1 and CDH13. 3D surface plots of staining intensity (represented in the middle panel of each row) while quantification of staining intensity in tumoroid (T) or invading cells (I) indicated as the percentage of cells that display stronger staining (represented in the third panel of each row). * p-value < 0.05; ns=not significant

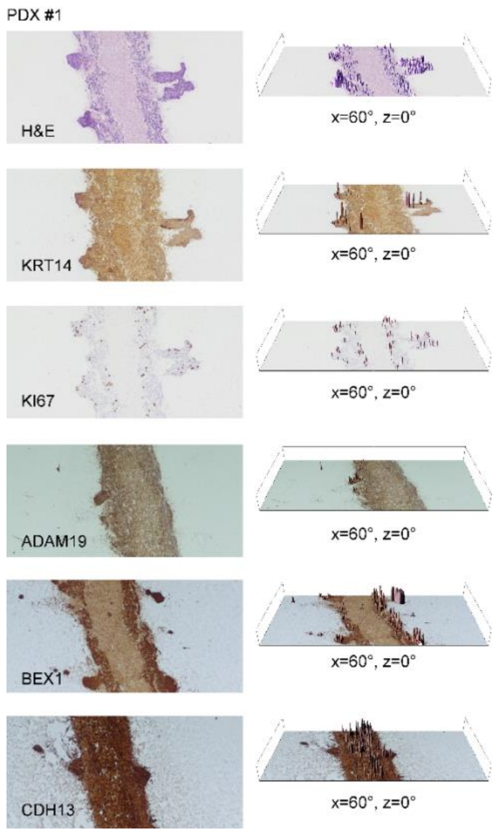

Supp. figure 4. Immunohistochemistry of PDX#1 tumoroids for markers of invasion. PDX #1 tumoroids were stained for KRT14, Ki67, ADAM19, BEX1 and CDH13.

Age	Sex/race	Type	Grade	Receptor status
53	Female/Caucasian	Invasive ductal carcinoma	3	Triple negative
55	Female/Caucasian	Invasive ductal carcinoma	3	Triple negative
53	Female/Caucasian	Invasive ductal carcinoma	3	Triple negative

Supplementary Table 1. PDX patient donor history. All three PDXs used were from patient donors with triple negative grade 3 invasive ductal carcinoma.

Pathway	Description	Count in gene set	False discovery rate
HSA-1474244	Extracellular matrix organization	13 of 298	0.0135
HSA-425393	Transport of inorganic cations/anions and amino acids/oligopeptides	7 of 104	0.0432

Supplemental table 2. Pathway analysis using STRING. Analysis of the 305 differential expressed genes using STRING yielded two Reactome pathways.
