# Supplementary material for: Heterogeneity at the invasion front of triple negative breast cancer cells
Source: Sci Rep. 2020 Apr 1;10:5781. doi: 10.1038/s41598-020-62516-8 (PMC7113246; doi:10.1038/s41598-020-62516-8)
Supplement: Supplementary file 2 — Supplementary Information. [file 41598_2020_62516_MOESM2_ESM.pdf]

Heterogeneity at the invasion front of triple negative breast cancer cells

Koh Meng Aw Yong[1,‡], Peter J. Ulintz[1], Sara Caceres[1,#] Xu Cheng[1], Liwei Bao[1], Zhifen Wu[1], Evelyn M. Jiagge[2], Sofia D. Merajver[1,*]

[1] Department of Internal Medicine, Hematology/Oncology University of Michigan Medical School, Ann Arbor, 48109; U.S.A.

‡ Koh Meng Aw Yong is currently at University of Michigan, Department of Urology, Ann Arbor 48109, U.S.A.

# Sara Caceres is currently at Department of Physiology, School of Animal Medicine. University Complutense of Madrid, Madrid, 28040, Spain.

[2] Evelyn M Jiagge is currently at the Henry Ford Cancer Institute/ Henry Ford Health System, One Ford Place, Detroit, Michigan.

* Corresponding author (smerajve@umich.edu)

Disclosures: The fluidic device used in this study for tumoroid culture has been licensed to K.A. (patent pending).

Supp. Video 1. SUM149 cells were cultured in fluidic device and images taken on days 0-11, 13-17, 19 and 21. Images were stacked into a time lapse using ImageJ.

Supp Video 2. MDA-MB-231 cells were cultured in the fluidic device and images taken on days 0-5 and 7. Images were next stacked into a time lapse using ImageJ.

Supp. Video 3. BT549 cells were cultured in the fluidic device and images taken on days 0-4, 7-18, 20 and 21. Images were stacked into a time lapse using ImageJ.

Supp. Video 4. HCC1937 cells were cultured in the fluidic device and images taken on days 0-3, 5-9. Images were stacked into a time lapse using ImageJ.

Supp. Video 5. PDX#1 cells were cultured in the fluidic device and images taken on days 0-1; 3; 5-7; 9; 11-15; 18-22; 25-34.

Supp. Video 6. PDX#2 cells were cultured in the fluidic device and images taken on days 0-1; 3-14; 16-21; 26-27; 30-31.
